# Incidental littoral cell angioma of the spleen

**DOI:** 10.1186/1477-7819-6-87

**Published:** 2008-08-19

**Authors:** May Tee, Patrick Vos, Peter Zetler, Sam M Wiseman

**Affiliations:** 1 Faculty of Medicine, University of British Columbia, Vancouver, British Columbia, Canada; 2 Department of Radiology, St. Paul's Hospital & University of British Columbia, Vancouver, British Columbia, Canada; 3 Department of Pathology & Laboratory Medicine, St. Paul's Hospital & University of British Columbia, Vancouver, British Columbia, Canada; 4 Department of Surgery, St. Paul's Hospital & University of British Columbia, Vancouver, British Columbia, Canada

## Abstract

**Background:**

Littoral cell angioma (LCA) is a recently described primary vascular neoplasm of the spleen that may be associated with other malignancies and may itself also have malignant potential.

**Case presentation:**

We present a case of LCA that was discovered incidentally in a 52-year-old woman who presented with biliary colic at the time of consultation for cholecystectomy. This vascular neoplasm was evaluated by ultrasound, CT, MRI, Tc-99m labelled red blood cell scintigraphy, and core biopsy. A splenectomy revealed LCA by pathological evaluation. Post-operative outcome was favourable with no evidence of complication or recurrent disease. Following this case presentation, clinical, radiographic, and pathological features of LCA will be reviewed as well as recent advances in our understanding of this uncommon splenic lesion.

**Conclusion:**

LCA is a rare, generally benign, primary vascular tumour of the spleen that typically is discovered incidentally. Individuals diagnosed with this tumour must be carefully evaluated to exclude primary, secondary, and synchronous malignancies.

## Background

Littoral Cell Angioma (LCA) of the spleen was recently described by Falk et al. in 1991 [1]. This group reviewed 200 surgical specimens of benign vascular splenic tumours and found 17 similar tumours that appeared related to the cells lining the red pulp splenic sinuses [1]. These tumours were unique in that they displayed both epithelial and histiocytic properties based on their cell of origin, the splenic littoral cells [1]. From these observations, this group designated this new vascular tumour of the spleen LCA [1].

Since this initial description, there have been scattered case reports and few case series of LCA [2-10]. The clinical presentation of LCA ranges from being completely asymptomatic and discovered incidentally, to presenting with a constellation of signs and symptoms such as abdominal pain, vague constitutional symptoms, splenomegaly, and hypersplenism [2-6]. Although first described as benign, LCA has recently been shown to exhibit malignant potential [11,12] and may also be associated with other visceral malignancies [13]. In this report, we present a case of LCA including its diagnostic work-up, surgical treatment, pathological evaluation, and post-operative outcome. A discussion regarding the clinical, radiological, and pathological features of LCA, as well recent advances in our understanding of this uncommon splenic tumour are also presented.

## Case presentation

**Figure 1:**
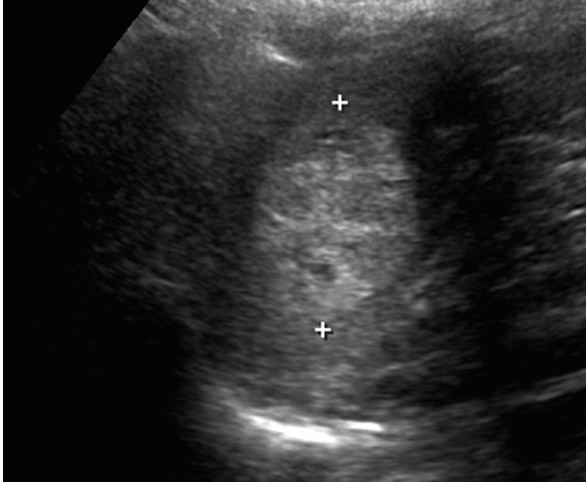
Ultrasound of the spleen demonstrates a well-defined hyperechoic lesion.

A 52-year-old woman was evaluated for symptoms of biliary colic for possible cholecystectomy. She described intermittent episodes of right upper quadrant pain with no history of jaundice, nausea, vomiting, or changes in bowel habits. Laboratory tests revealed elevated liver enzymes of a cholestatic nature but total bilirubin within normal limits (ALP = 139 U/L, GGT = 67 U/L, total bilirubin = 6 μmol/L). Haemoglobin, white blood cells, and platelet counts were within normal limits. Ultrasound (US) revealed cholelithiasis, a normal appearing biliary tree, and fatty infiltration of the liver. Notably, US also identified a hyperechoic, well-circumscribed, 3 cm lesion located at the inferior aspect of the spleen (figure 1). No significant vascularity was noted on the color Doppler images.

**Figure 2:**
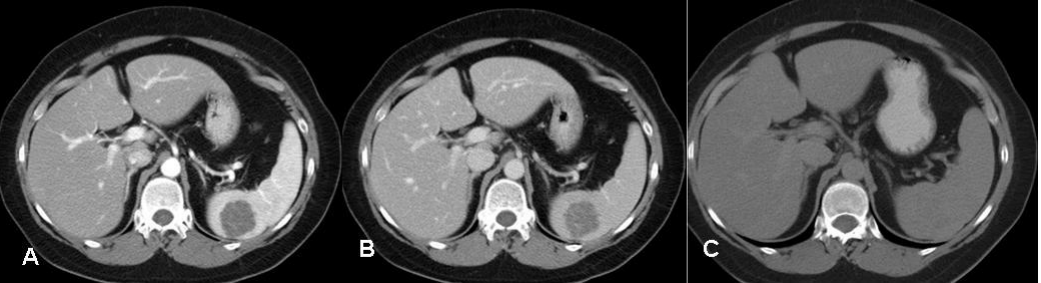
CT scan after oral and iv-contrast in the arterial phase **A **and portal venous phase **B **demonstrates a hypodense well defined round lesion in the posterior portion of the spleen with some linear internal enhancement. **C**. The lesion is isodense compared to the normal spleen on the 5 minutes delayed image.

**Figure 3:**
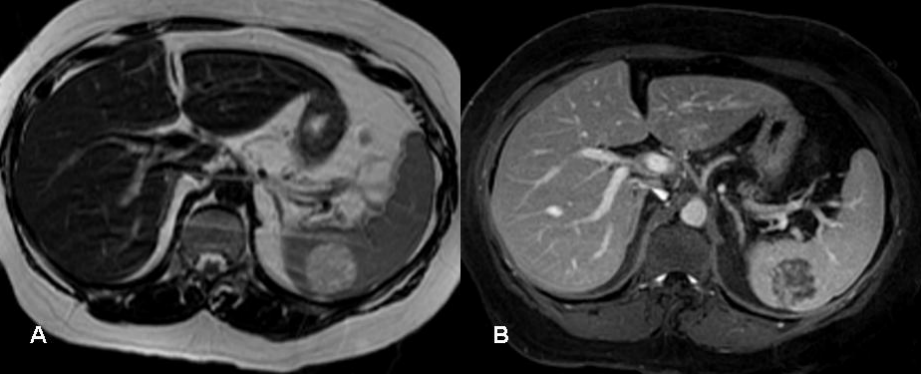
**A**. The lesion is hyperintense on the T2 weighted fast recovery fast spin echo (FRFSE) image. **B**. Internal linear enhancement is noted on the T1 weighted fat saturated image after iv-gadolinium in the portal venous phase.

The splenic lesion was further evaluated with CT, MRI. A hypodense well defined lesion with some internal enhancement in the arterial and venous phases was demonstrated on the contrast enhanced CT scan (Fig. 2A, 2B). The lesion was isodense compared to the surrounding splenic parenchyma on the 5 minutes delayed images (Fig. 2C). On MR, the lesion was hypointense on the T1, and hyperintense on the T2-weighted sequences (Fig. 3A). After the administration of IV gadolinium the lesion demonstrated some internal linear enhancement in the portal venous phase on the T1-weighted fat suppressed sequence (Fig. 3B) and became isointense on the delayed images. A Tc-99m labelled red blood cell scan showed the splenic lesion to be 'cold'. A percutaneous ultrasound guided core biopsy of the lesion was subsequently carried out but was nondiagnostic as histological evaluation showed skeletal muscle. Overall, it was felt that the splenic lesion had a benign appearance and would be followed up with imaging.

A laparoscopic cholecystectomy was performed. No splenic lesion was grossly evident at the time of laparoscopy. The postoperative course was uneventful and episodes of abdominal pain resolved post-operatively. A colonoscopy was also performed, which was normal. A repeat CT scan 6 months postoperatively showed interval enlargement of the splenic lesion as well as the development of an adjacent satellite lesion with a similar radiological appearance. As a result of the enlargement of the lesion, and development of a new lesion, a decision was made to carry out a splenectomy.

**Figure 4:**
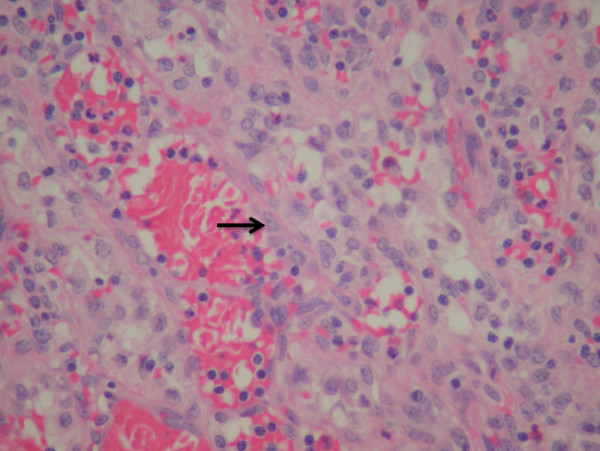
Anastamosing vascular channels lined by plump cells with the appearance of sinus lining (arrow: 'littoral' cells) (H&E 100×).

At the time of open splenectomy, the spleen was unremarkable in terms of size and appearance, and was removed in its entirety for pathological evaluation. This patient had an uneventful post-operative recovery and has remained well at fifteen months of post-operative follow-up. Pathologic evaluation identified a non-encapsulated but well-circumscribed reddish nodule that was 4 × 3 × 3 cm in size and located at the anterior-inferior pole of the spleen. Histologically, this lesion was described as a vascular neoplasm forming anastomosing vascular channels lined by histiocytes with occasional papillary structure, consistent with Littoral Cell Angioma (Fig. 4).

## Discussion

Primary vascular tumours of the spleen are uncommon but represent the majority of non-hematolymphoid splenic tumours [14]. The differential diagnosis of splenic vascular tumours is broad and may represent benign (haemangioma, haemartoma, lymphangioma), indeterminate (littoral cell angioma, haemangioendothelioma, haemangiopericytoma), or malignant neoplasms (angiosarcoma) [14]. LCA is a recently described vascular tumour of the spleen that is now classified as having uncertain biological behaviour, given several case reports which have identified malignant potential [14].

The exact incidence of LCA is unknown although the incidence of splenic haemangioma varies from 0.03% to as high as 14% in one reported autopsy series [15]. LCA does not have any particular gender or age predilection although the median age in Falk *et al*.,'s original study of LCA was 49 years [1,14]. As was the case for our patient, LCA may be completely asymptomatic and represent an incidental finding by imaging [13,16]. LCA may also present with a myriad of possible signs and symptoms, such as: splenomegaly with or without abdominal pain, hypersplenism with ensuing anaemia and/or thrombocytopenia, and constitutional symptoms such as intermittent fevers. More dramatically, LCA has been reported to present as splenic rupture and haemoperitoneum [13-15].

LCAs are believed to originate from the littoral cells, the cells that line red pulp sinuses of the spleen [1,17]. From studies performed as far back as the 1930s, endothelial cells lining the vascular sinuses of the spleen were considered unique in that they exhibited both phagocytic and hematopoietic properties [10]. Neoplasia of these cells results in the formation of LCA, which exhibit histologic and molecular features consistent with both these epithelial and histiocytic cell types [17,18].

The pathogenesis of LCA remains unclear, but given its association with autoimmune disorders such as Crohn's disease and inborn metabolic diseases such as Gaucher's disease, immune system dysfunction has been postulated as a possible important pathogenic mechanism [10,19]. Supporting this hypothesis, other reports have suggested that chronic infection and systemic immunosuppression may contribute to LCA development [13,18].

Indeed, immune system dysregulation may explain the association of LCA with other cancer types. The other cancer types associated with LCA include: thyroid, colorectal, renal, pancreatic, hematologic (lymphoma), ovarian, and testicular cancer [10,13,18,20,21]. These observations have prompted recommendations to closely evaluate and provide surveillance to patients with LCA for the development of other malignancies [20]. Conversely, the association of LCA with other cancer types may also be a result of making an incidental diagnosis of LCA during extensive radiological imaging for other diseases, given the largely asymptomatic presentation of these tumours [22].

However, close follow-up of LCA may be warranted due to the potential for their malignant transformation. The two subtypes of LCA with malignant potential have been described as "littoral cell angiosarcoma" [18,23,24] and "littoral cell haemangioendothelioma" [11,12,18]. These LCA variants may present with distant metastasis several months after splenectomy; histologic evaluation reveals features consistent with LCA histopathology as well as abnormal architecture, nuclear atypia, and necrosis [11,12,23,24].

Radiologically, LCA may be evaluated by several imaging modalities such as US, CT, MRI, or nuclear medicine studies (Tc-99m labelled RBC scintigraphy). US may reveal lobular splenomegaly with heterogeneous nodules (either hypo- or hyper-echoic) that may be solitary or multiple (Figure 1) [25]. On non-contrast CT, LCA appear as hypo-attenuating masses; given the vascular nature of these neoplasms, they tend to enhance homogeneously. On MRI, a minority of cases may be hypointense on both T1-weighted and T2-weighted scans because of hemosiderin content of the tumour [14]. However, as significant siderosis is seen in less than 50% of LCA cases [1], lesions tend to be hyperintense on the T2 weighted images, as was the case in our patient (Figure 3) [26]. Nuclear medicine studies with Tc-99m labelled RBC scintigraphy can be useful to differentiate splenic lesions from splenic haemangiomas [27,28]. However, the radiologic features of LCA are rarely diagnostic since many other splenic neoplasms such as haemartomas, haemangiomas, lymphomas, metastatic disease and infectious processes exhibit similar imaging characteristics [29].

Gross pathology of LCA is characterized by single, or more commonly, multiple pigmented focal nodules well-delineated from normal spleen parenchyma [28,30]. The colour of these nodules may be dark red, brown, or black, consistent with blood or blood products of varying chronicity [28]. Rarely, LCA appears white on gross pathology [1,30]. The size of these lesions varies and may range from 0.1 cm to 11 cm in diameter [1,30,31]. The spleen itself may appear grossly enlarged or, as was true for our case, look otherwise unremarkable [31,32].

Microscopically, there are several distinguishing histological and molecular features of LCA. Histologically, LCA has specific features that differentiate it from other primary vascular tumours, including angiosarcoma [1]. LCA are composed of anastomosing vascular channels resembling splenic sinusoids and have irregular lumina featuring papillary projections and cyst-like spaces (Figure 4) [1]. Tall endothelial cells with histiocytic properties that slough off into the vascular lumen are common, as is the absence of atypical cells and presence of low mitotic activity [1]. By immunohistochemical staining, these tumour cells will express endothelial and histiocyte antigens, a reflection of the distinct dual differentiation potential of LCA [1]. Such expression includes endothelial markers (factor VIII Ag and CD 31/BMA 120) as well as histiocytic markers (CD 68/KP 1 and lysozyme) [1,4,30,31,33]. The expression of these molecular markers has also been demonstrated in fine-needle aspiration biopsies of LCA [33].

Symptomatic LCA are often relieved by splenectomy, and given the association of LCA with other malignancies and reported cases of metastasizing LCA, splenectomy is both diagnostic and therapeutic. While there have been reports of medical therapy with glucocorticoids and angioembolization of splenic haemangiomas [34], splenectomy is still considered the gold standard for treatment of vascular splenic tumours [15].

## Conclusion

LCA is a recently described primary vascular neoplasm of the spleen that may be associated with other malignancies and may itself also have malignant potential. Several radiological studies may suggest LCA, although a pathological diagnosis, either by core biopsy or diagnostic splenectomy is imperative. This rare case illustrates the importance of thoroughly evaluating incidental vascular splenic tumours. Although the vast majority of LCAs are benign, their differential diagnosis must include both primary and secondary malignancy, given LCA's association with other cancer types as well as their uncertain malignant potential. With this in mind, gold standard management remains splenectomy and long-term follow-up for the development of synchronous tumours or metastatic lesions is advised.

## Competing interests

The authors declare that they have no competing interests.

## Authors' contributions

MT performed the literature review and drafted the manuscript. PV reviewed and revised the manuscript and provided radiographic images. PZ reviewed and revised the manuscript and provided pathologic images. SW originated the idea and assisted with drafting and revising the manuscript. All authors read and approved the final manuscript.
